# Public perceptions of mental health and the role of nursing professionals in providing psychological support: a nation-wide, cross-sectional study from Croatia

**DOI:** 10.1192/j.eurpsy.2025.1611

**Published:** 2025-08-26

**Authors:** T. Meštrović, T. Ćurić, M. Neuberg, I. Herak, R. Ribić

**Affiliations:** 1 Department of Nursing, University North, Varaždin, Croatia

## Abstract

**Introduction:**

Mental health is indispensable to quality of life and social well-being, influencing economic stability, human rights and sustainable development. Despite growing awareness, the public often conflates mental health with mental illness, which means that stigma remains prevalent. Nursing professionals, who interact closely with patients, are uniquely positioned to provide psychological support. Therefore, understanding public perceptions of mental health and the role of nurses is crucial for developing effective care strategies and improving health outcomes.

**Objectives:**

With our research we aimed to examine public attitudes towards mental health and assess how the general population perceives nurses’ roles in psychological support. The study also sought to identify demographic influences on these attitudes and highlight potential improvements in nursing education.

**Methods:**

The study utilized a cross-sectional survey design to collect quantitative data on public perceptions of mental health and the roles of nursing professionals. An anonymous online questionnaire was distributed, reaching a diverse sample of 270 participants from various demographic backgrounds. The survey included items assessing demographic information (gender, age, educational level, and residential location), as well as specific questions on mental health perceptions, experiences with psychological challenges, and also attitudes toward nursing empathy and support. Descriptive and inferential statistics - including Mann-Whitney U tests and chi-square tests - were used to analyze responses. Significance was set at p<0.05.

**Results:**

Of the respondents, 73% were female, 63.3% resided in urban areas, and 54% had completed secondary education. The majority (65%) were over 35 years old. Regarding self-assessed mental health, 48.9% rated it as “good,” and 48.5% reported occasional mental health challenges. Significant gender differences were observed in perceptions of empathy among nursing professionals, with female respondents reporting more positive views compared to male respondents (U=83.37, p=0.01). The study also revealed that 53% of participants believed nurses “sometimes” showed empathy toward patients’ mental health needs. A significant association was found between educational level and the perception that nurses need additional training to support mental health challenges (U=147.00, p=0.01).

**Conclusions:**

This study highlights the vital role of nurses in mental health support and suggests that additional training on psychological support could enhance care quality, especially in the eyes of well-educated patients. While many respondents see nursing professionals as empathetic, there is room to further improve nurses’ mental health literacy and communication skills to reduce stigma and improve patient outcomes.

**Disclosure of Interest:**

None Declared

